# The Hospital Dietary

**Published:** 1916-12-09

**Authors:** Elliott P. Joslin

**Affiliations:** Associate Professor of Medicine, Harvard University, Boston.


### THE HOSPITAL DIETARY.*

### II.—Educational Aspects.

By Dr. ELLIOTT P. JOSLIN, Associate Professor of Medicine, Harvard University, Boston.

THE educational aspects of the dietary in a hospital deserve attention. From the administrative point of view first, all emphasis should be laid upon the necessity for the better training of dieticians. Too often, when changes are made in the hospital dietetic staff, months are lost because the new incumbent lacks experience. Dieticians should have a period of service in hospitals quite the same as medical students and house officers. It is quite as essential for dieticians to have practical experience and to bear responsibility under supervision in the arrangement of diets as it is for house officers to be given training and responsibility in the care of patients.

Nurses are already given courses in dietetics, but the need for more instruction is daily apparent, because treatment in hospitals more and more is based upon quantitative variations in the diet. Experience alone will enable nurses to carry out orders properly. Nurses may know more about diets than the older physicians, but they do not as a rule know more than the present younger group of doctors. All nurses working in institutions should have demonstrated to them the necessity for the avoidance of waste. They should see a garbage inspection and be shown in this way how useful they can be to their institution. In order to familiarise them with the caloric value of foods, each nurse's dining-room should have a "calorie" table, and a group of nurses should be assigned to it for a week and calculate the values of the foods they eat. The students adopted this plan at their lunch-room in the Harvard Medical School with profit. Practical instruction of this type is interesting, stimulating, and costs nothing.

Students require more instruction in dietetics than they now receive. It is for the interest of hospitals to encourage their interest in patient's diets, to encourage their asking questions and to bring out their criticisms. Students will talk when house officers are afraid to express opinions.

By this means many old-fashioned notions will be eliminated from the dietary. They, too, should see a garbage inspection.

Finally, the hospital staff should have presented to it in brief form each year some of the important administrative problems which come up to the superintendent, and foremost among these is the dietetic problem. Is not the staff perhaps too often told not to do this or that, whereas if assembled in a body and reasons for the innovation proposed were set forth, its sympathy with new methods would be easily gained. It is easier to lead than to drive.

Most of all, the patients require instruction in the diet. No patient should go out of the hospital without having had explained in one way or another the advantages of the diet given. His stay in the hospital should be a course in hygiene. With most cases this would be in the nature of an explanation of how to procure simple foods at low cost, but with others detailed instruction should be given. The subject should not be left until the last day, but the important points of diet should be systematically taught throughout his stay. Medicines are easy to take, but the reason for special diets are easily forgotten. If a patient requires a diet for constipation, he should be given such a diet in the hospital, and its character should be explained so many times that he will not forget it. The diets for patients with diseases of the stomach and intestines are not complicated, but the reasons for their peculiarities are often not appreciated. With diabetes, diet, except in very mild cases, is everything, and in this disease the stay of the patient in the hospital is almost exclusively for the purpose of being taught dietetics. The whole aim in the modern treatment of diabetes is not to render the urine sugar-free, but rather to so instruct the patient that he can keep it so when at home. In the treatment of diabetes, not only the patient but the relatives must receive instruction, for they will care for the patients later on. Patients learn readily, and children more so than adults. Indeed, in the kitchen at the Deaconess Hospital more than one little girl has acquired the art of cooking, and I always feel comparatively safe in sending home a twelve-year-old child upon a diet, because I am quite sure it will be followed. This instruction of patients' relatives is not a new procedure, for the standard rules in your out-patient departments for the feeding of children are not only specific, but designed to instruct the mother as well. Furthermore, I was interested to see that the directions for stomach cases in the out-patient department of the Massachusetts General Hospital contain few specific statements about diet, but such excellent general advice that, if followed, indigestion must disappear.

The diet of each diabetic patient is prescribed in simple terms, and not uncommonly fifty to a hundred words cover all the orders given a patient for a three-weeks' stay. Even these are written in abbreviations. The problem is simplified because diabetic patients eat chiefly eggs, meat, bacon, fish, oil, cream, and groups of vegetables containing 5, 10, and 15 per cent. carbohydrate. How very simple the matter is will be seen by inspecting one of the daily charts. These charts are simply large blocks of newspaper paper, and it will be seen that the diet for ten patients for two meals occupies very little space. The charts hang in the kitchen, and Miss Smith, who is in charge of this portion of the hospital, usually weighs out the food herself, and the two nurses who aid her in the care of the seventeen patients, with the help of the patients, distribute the trays. With each tray goes a little card, speci-

* A Paper read at the American Hospital Conference at Philadelphia, September 1916. For the first part see THE HOSPITAL, December 2, 1916, page 181.

fying the amounts of the various foods served. At the end of the day these cards are collected, the amounts of the various foods are added together by the patient, and the content of carbohydrate, protein, and fat reckoned. New nurses find it complicated and so do new patients, but I do not remember a twelve-year-old child who has been unable to reckon up his diet. In fact, usually the children are used as teachers of the newcomers, and it is sometimes quite amusing to see a twelve-year-old child teaching a patient of sixty. It does no harm for the patients to reckon up their diets each day, although it may be unnecessary for them to do it outside.

Patients are welcomed in the kitchen on invitation, are deliberately exposed to temptation, and special arrangements are made to have patients' relatives come to the hospital to learn how to prepare the food. To lessen cost, patients' services must be utilised. Europe will teach us many lessons in this regard. I hope for a diabetic patients' vegetable, not flower, garden. A few nurses have spent a week, paying their own board and volunteering their services, at the hospital for the knowledge gained. They have an opportunity of seeing the cases and of learning how to arrange their diets, test their urines, and how to make these patients comfortable while going through the treatment. By seeing a group of patients nurses cease to be afraid to carry out rigid treatment with individuals. Then, too, they usually see some cases who are doing quite well, have an opportunity to see cases who have been under treatment for several years, and by this means are encouraged to persist in the rigorous treatment of severe cases, to which they are usually sent. As you are aware, the demand for good diabetic nurses considerably exceeds the supply, and it is a type of work peculiarly attractive to a nurse who has a scientific bent, enjoys accuracy, and likes to exercise her mind.

Co-operation.

Co-operation in hospitals between superintendents, nurses, the medical, surgical, and pathological staffs, should be more intimate. Too often the horizon of each has been limited by his own department. The same practice rules elsewhere, in fact is universal, because it is human nature, but lessons in co-operation are now being taught the world on every side. We must not be behind in this movement. You have kindly asked me to speak to you instead of listening to you, but I shall not forget to reverse the status, and our medical societies will gain in the exchange.

